# Increased Toll-Like Receptor 2 Expression in Peptidoglycan-Treated Blood Monocytes Is Associated with Insulin Resistance in Patients with Nondiabetic Rheumatoid Arthritis

**DOI:** 10.1155/2012/690525

**Published:** 2012-11-20

**Authors:** Shih-Wei Wang, Tsun-Mei Lin, Chiou-Huey Wang, Hsiao-Han Liu, Jer-Yiing Houng

**Affiliations:** ^1^Division of Allergy, Immunology, and Rheumatology, Department of Internal Medicine, E-DA Hospital, No. 1 Yida Road, Yanchao District, Kaohsiung 82445, Taiwan; ^2^Institute of Biotechnology and Chemical Engineering, I-Shou University, Section 1, No. 1 Syuecheng Road, Dashu District, Kaohsiung 84001, Taiwan; ^3^Department of Medical Laboratory, E-DA Hospital and I-Shou University, No. 1 Yida Road, Yanchao District, Kaohsiung 82445, Taiwan; ^4^Department of Medical Research, E-DA Hospital and I-Shou University, No. 1 Yida Road, Yanchao District, Kaohsiung 82445, Taiwan; ^5^Graduate Institute of Medicine, College of Medicine, Kaohsiung Medical University, No. 100 Shih-Chuan 1st Road, Kaohsiung 80761, Taiwan; ^6^Department of Biological Science and Technology, I-Shou University, No. 8 Yida Road, Yanchao District, Kaohsiung 82445, Taiwan; ^7^Department of Nutrition, I-Shou University, No. 8 Yida Road, Yanchao District, Kaohsiung 82445, Taiwan

## Abstract

The close relationship between increased TLR-2 expression in blood monocytes and insulin resistance in RA patients is shown in this study. Traditional risk factors for metabolic disorders, including the waist circumstance, body mass index (BMI), triglyceride (TG), and ratio of TG to high density lipoprotein (HDL) cholesterol, were closely correlated with HOMA (homoeostasis model assessment) index in patients with nondiabetic RA. Expressions of TLR2 in peripheral blood monocytes, following stimulation with peptidoglycan which is known as a TLR2 agonist, were closely correlated with the HOMA index, TNF-**α**, and IL-6 concentrations. Accordingly, TLR-2 receptor and its related inflammatory cytokines could be potential therapeutic targets in managing insulin resistance in RA patients.

## 1. Introduction

Rheumatoid arthritis (RA) is a complex disease whose pathogenesis remains unknown. Patients with RA have systemic inflammation, as well as increased morbidity and mortality from cardiovascular disease [1, 2]. Various risk factors have been identified that contribute to atherosclerosis in RA patients [3, 4]. Insulin resistance is the most important of these risk factors. Recent years have seen increased attention devoted to inflammation associated insulin resistance [5]. Increasing numbers of research studies illuminate several possible mechanisms, including activation of innate system. 

Toll-like receptors (TLRs), as key molecular components of the innate immune system, are of a central interest in innate system associated insulin resistance. Twelve members of TLRs have been identified in mammals [6]. The predominant site of TLRs expression is on cells of the innate system, particularly on monocytes [7]. Monocytes are involved in inflammation and the development of insulin resistance [8–10]. The TLRs recognize numerous ligands; for example, TLR2 and TLR4 were hypothesized to recognize components of the bacterial cell wall such as peptidoglycan and lipopolysaccharide, respectively, and to interact with lipid-containing molecules [11]. TLRs participate in the pathogenesis of insulin resistance in animal models [12, 13] and mediate vascular inflammation and insulin resistance in diet-induced obesity [14]. Additionally, TLRs can link innate immunity and fatty acid-induced insulin resistance [12]. Activation of TLRs in adipocyte has been implicated in the onset of insulin resistance in obesity and type-2 diabetes [15]. Raised TLR expression and signaling were also observed in muscles of insulin resistant individuals [16]. High glucose can induce TLR2 and TLR4 expression, activity, and inflammation via nuclear factor (NF)-*κ*B [17]. Dasu and Jialal further reported that free fatty acids in the presence of high glucose may amplify monocyte inflammation through TLRs [18]. Saturated fatty acid may serve as a ligand for several members of the TLR family [19, 20]. Furthermore, TLR2, following ligation with specific ligands, can activate signal transduction, promote interleukin (IL)-6 production, and mediate initial events related to fatty acid-induced insulin resistance in muscle [21]. Tumor necrosis factor-alpha (TNF-*α*) stimulation was also suggested to disrupt insulin signal transduction and induce insulin resistance [22]. Additionally, the inflammatory kinase I-kappa-B kinase-*β* (Ik*κβ*) contributes to insulin resistance by activating NF-*κ*B and induces production of various inflammatory cytokines, including TNF-*α* and IL-6 [23, 24]. TLRs thus might modulate inflammation and insulin resistance following ligation with specific ligands. Although numerous studies have demonstrated that TLR2- and TLR4-dependent signaling are involved in the development of insulin resistance, data on a similar mechanism in the pathogenesis of insulin resistance in RA patients are still lacking.

TLRs activation has been described as being involved in the pathogenesis of RA, and both TLR2 and TLR4 are potentially important receptors in the initiation and perpetuation of the inflammatory cycle in arthritis [25]. TLRs are present on tissue synoviocytes and blood monocytes which are recruited to the site of inflammation and involved in the pathogenesis of synovial inflammation [26–29]. Thus, TLRs-inducing inflammatory cascades potentially can contribute to the pathogenesis of insulin resistance in RA patients. However, the evidence of the relationship between TLRs and insulin resistance in RA patients remains rare. This investigation tests the hypothesis that expressions of TLR2 or TLR4 on monocytes and related inflammatory cytokines, such as TNF-*α* and IL-6, might be associated with insulin resistance in patients with RA. Notably, this study excluded RA patients with diabetes mellitus. TLR2 still plays a role in the development of insulin resistance in patients with RA even in the absence of hyperglycemia, which is a well-known risk factor for metabolic syndrome.

## 2. Materials and Methods

### 2.1. Study Design and Subjects

The study population included 30 consecutive RA patients that fulfilled the American College of Rheumatology (ACR) 1987 classification criteria [30] and 10 healthy volunteers. Patients or normal controls with diabetes mellitus were excluded. Written informed consents were obtained from the patients before enrollment. The study was in agreement with the guidelines approved by the Human Research Ethics Committee at our hospital. Demographic data, clinical characteristics, and current medications of the patients were recorded by two independent observers.

### 2.2. Assessments

Waist circumference was measured at the umbilical level. Overnight fasting blood samples were taken to determine blood glucose, serum insulin level, triglyceride (TG), and cholesterol profiles including total cholesterol, LDL cholesterol, and HDL cholesterol. High sensitivity C-reactive protein and Westergren erythrocyte sedimentation rates were determined at the E-DA Hospital Clinical Laboratory. RA disease activity was measured using the Disease Activity Score in 28 joints. To measure the insulin resistance, the homoeostasis model assessment (HOMA), as described by Matthews et al. [31], was calculated using the formula: ([fasting plasma glucose (mmol/L) × fasting plasma insulin (*μ*U/mL)]/22.5). Monocyte staining for TLR2 and TLR4 expression was performed on the whole blood of RA patients and healthy controls before and stimulation with peptidoglycan from *Staphylococcus aureus* (10 *μ*g/mL, Sigma-Aldrich, MO, USA) or lipopolysaccharide from *E. coli* O26 : B6 (10 ng/mL, Sigma-Aldrich) as ligands for TLR2 and TLR4. Whole blood specimens were incubated with antibodies against TLR2 (eBioscience, CA, USA) or TLR4 (PE labeled, BioLegend) and anti-CD14 conjugated with FITC (BD Biosciences, CA, USA) for 30 min at room temperature in the Polystyrene Round-Bottom Falcon Tube (BD Biosciences). Appropriate isotype controls were also used. Two mL working 1X BD FACSTM Lysing Solution was added to the reaction tube for 10 min at room temperature to lysis red blood cells, and the excess unbound antibody was then washed with PBS (phosphate-buffer saline). Cells were finally resuspended in PBS and analyzed with a flow cytometer (Becton-Dickinson Immunocytometry Systems, San Jose, CA, USA) by counting 10,000 cells. Expressions of TLRs were calculated as mean fluorescence intensity (MFI) and percentage of CD14^+^ monocytes expressing TLR2 or TLR4 using WinMDI98 software (BD Biosciences). Whole blood samples were collected from both patients with RA and healthy volunteers via venipuncture into heparin containing tubes and diluted with 1X HBSS buffer at a 1 : 1 ratio. The blood samples were incubated in the presence of 5% CO_2_ at 37°C in 24-well plates using 10 *μ*g/mL peptidoglycan or 1 ng/mL lipopolysaccharide or medium alone. The cells were then pelleted via centrifugation (400 ×g for 2 min), and the cell-free supernatants were stored at −70°C for cytokine determination. Concentrations of IL-6 and TNF-*α* of cell supernatants were determined using ELISA kits (Bender MedSystems, CA, USA).

### 2.3. Statistical Analysis

All analyses were performed using the SPSS statistical software (version 15.0; SPSS Inc., Chicago, IL, USA). Chi-square test or Fisher's exact test, when necessary, was used for categorical values and Wilcoxon's rank sum tests for continuous variables. Spearman's rank correlation was calculated to assess relationships between variables. Only *P* values less than 0.05 were considered significant.

## 3. Results

### 3.1. Demographic and Clinical Characteristics of Normal Controls and RA Patients

**Figure 1:**
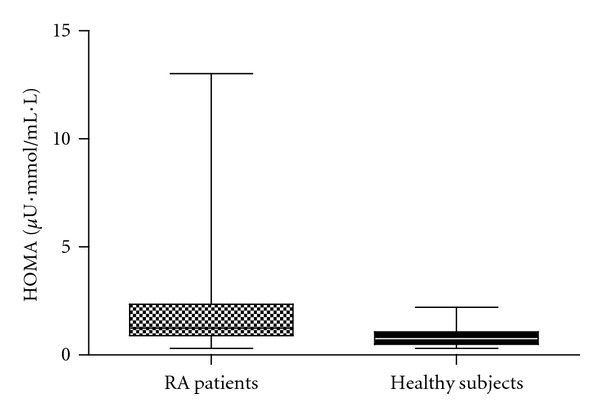
Homeostasis model assessment (HOMA) index in serum samples. Box plot graphs show HOMA index in serum samples from the 30 patients with rheumatoid arthritis (RA) and 10 healthy subjects. The *P* value was 0.017.

A total of 30 patients with RA (25 females and 5 males) aged 36 to 82 years were enrolled and 10 healthy controls aged 51 to 64 years were also recruited. Demographic characteristics, lipid profiles, cardiovascular risk factors, and the HOMA index for RA patients and normal controls are presented in Table 1. Age, sex, systolic blood pressure, diastolic blood pressure, total cholesterol, low-density lipoprotein, high-density lipoprotein, triglycerides, BMI, and blood glucose between groups showed no significant differences. HOMA index was significantly higher in RA patients than in healthy subjects (RA patients versus healthy subjects *P* = 0.017, Figure 1). Eighty-three percent of RA patients are current users of corticosteroid, but the cumulative dose of steroid showed no significant correlation with insulin resistance of these patients (*r* = 0.081, *P* = 0.675). The datum suggests that perhaps better control of inflammation after receiving treatment with steroid may counterbalance the deleterious effect of corticosteroids on glucose metabolism. Additionally, as would be expected, HSCRP (high sensitivity C-reactive protein) and ESR (erythrocyte sedimentation rate) revealed significantly higher concentrations in RA patients compared with normal controls (*P* = 0.008 and *P* = 0.002, resp.).

### 3.2. Relationship between HOMA Index and Waist, BMI, TG, and Ratio of TG to HDL Cholesterol in Patients with Nondiabetic RA

**Figure 2:**
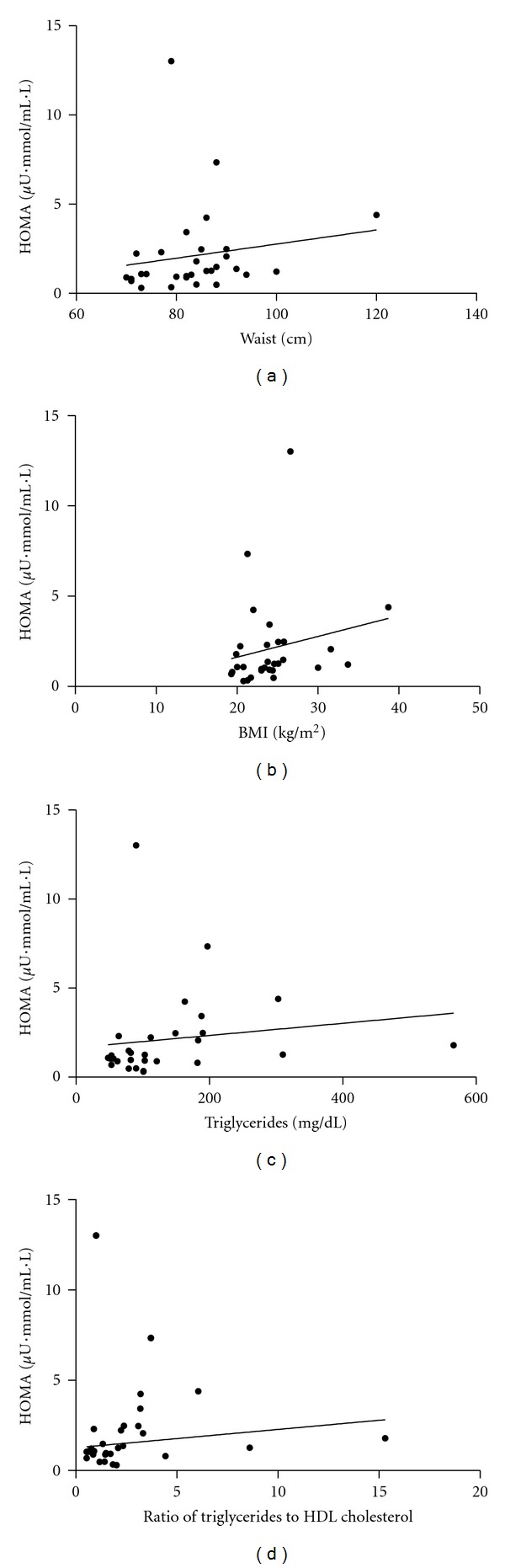
Relationship between HOMA index and waist (a), BMI (b), TG (c), and ratio of TG to HDL cholesterol (d) in patients with nondiabetic RA. In nondiabetic RA patients, HOMA index was significantly correlated with (a) waist (*r* = 0.381, *P* = 0.038), (b) BMI (*r* = 0.374, *P* = 0.042), (c) TG (*r* = 0.444, *P* = 0.014), and (d) ratio of TG to HDL cholesterol (*r* = 0.423, *P* = 0.020).

HOMA index was significantly correlated with waist (*r* = 0.381, *P* = 0.038), BMI (*r* = 0.374, *P* = 0.042), TG (*r* = 0.444, *P* = 0.014), and ratio of TG to HDL cholesterol (*r* = 0.423, *P* = 0.020) in nondiabetic RA patients (Figure 2). However, HOMA index was not correlated with total cholesterol (*r* = 0.076, *P* = 0.689) and LDL cholesterol (*r* = 0.093, *P* = 0.626) in RA patients. These results imply that monitoring traditional risk factors might also be important in managing insulin resistance in RA patients, even in the absence of hyperglycemia.

### 3.3. Relationship between HOMA Index and TLR2 Expression in Monocytes of Normal Controls and RA Patients

**Figure 3:**
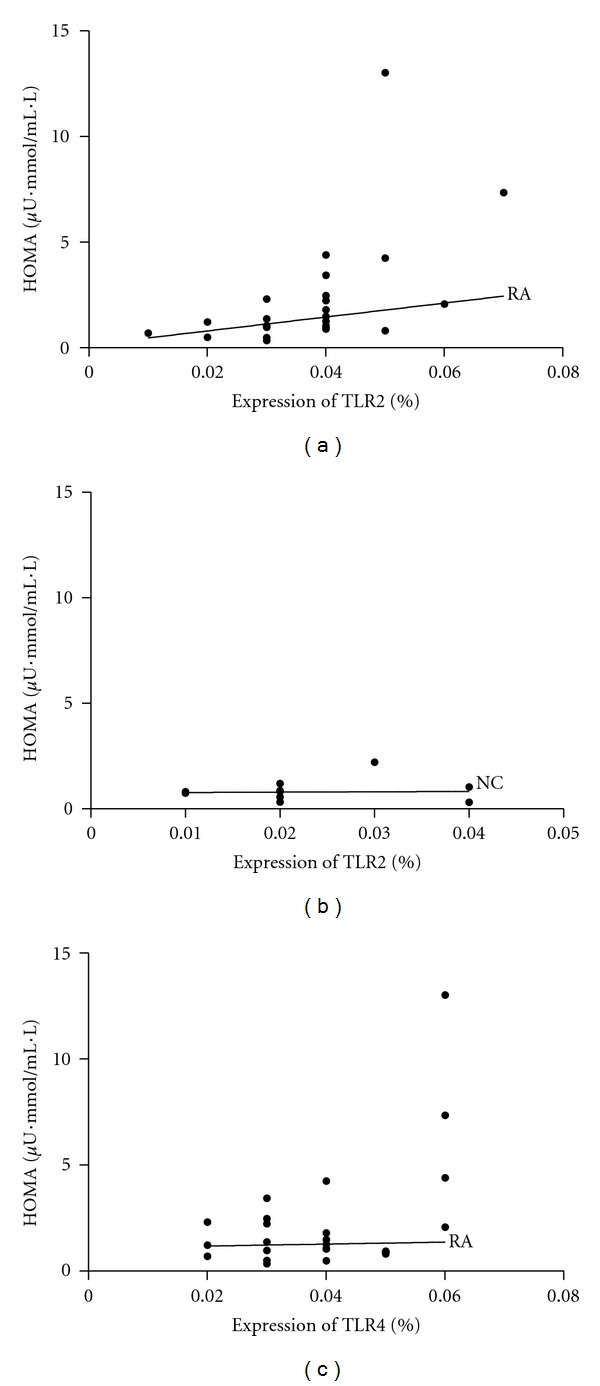
Relationship between HOMA index and TLR2 or TLR4 expression (calculated as percentage of CD14^+^ monocytes expressing TLR2 or TLR4) in monocytes after stimulation with peptidoglycan or lipopolysaccharide in RA patients and normal controls (NC). (a) Positive correlation between HOMA index and TLR2 expression after stimulation with 10 *μ*g/mL of peptidoglycan in RA patients (*r* = 0.514, *P* = 0.009); (b) no significant correlation between HOMA index and TLR2 expression in normal controls (*r* = 0.150, *P* = 0.682); (c) no significant correlation between HOMA index and TLR4 expression after stimulation with 10 ng/mL of lipopolysaccharide in RA patients (*r* = 0.230, *P* = 0.268).

As shown in Figure 3, HOMA index was significantly correlated with TLR2 expression (calculated as percentage of CD14^+^ monocytes expressing TLR2) after stimulation with 10 *μ*g/mL of peptidoglycan (*r* = 0.514, *P* = 0.009, Figure 3(a)), but not with TLR2 expressions before stimulation by peptidoglycan (*r* = 0.387, *P* = 0.056). In contrast, HOMA index was not significantly correlated with TLR2 expression in monocytes after stimulation with 10 *μ*g/mL of peptidoglycan in normal controls (*r* = 0.150, *P* = 0.682, Figure 3(b)). The experimental results revealed no significant correlation between HOMA index and TLR4 expression in monocytes before (*r* = 0.288, *P* = 0.162) or stimulation with 10 ng/mL lipopolysaccharide (*r* = 0.230, *P* = 0.268, Figure 3(c)).

**Figure 4:**
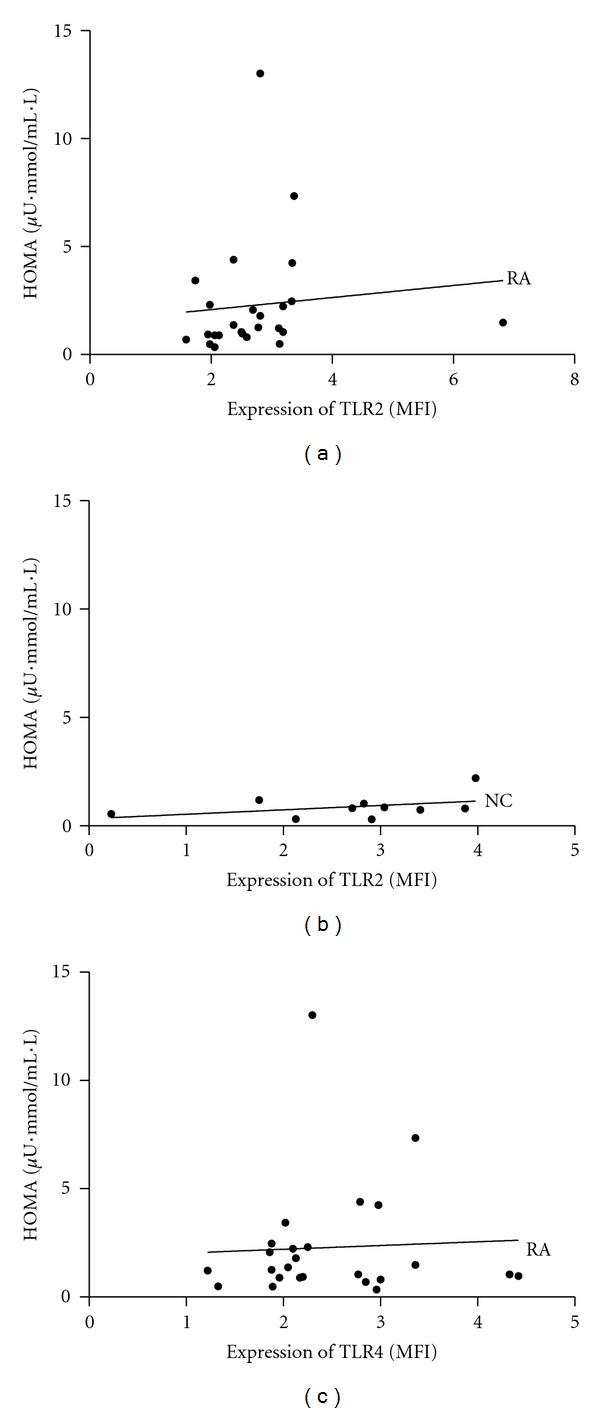
Relationship between HOMA index and fold increase of TLR2 or TLR4 expression (MFI) in monocytes after stimulation with peptidoglycan or lipopolysaccharide in RA patients and normal controls (NC). (a) HOMA index significantly correlated with TLR2 expression after stimulation with 10 *μ*g/mL of peptidoglycan in RA patients (*r* = 0.441, *P* = 0.027); (b) no significant correlation between HOMA index and TLR2 expression in normal controls (*r* = 0.249, *P* = 0.492); (c) no significant correlation between HOMA index and TLR4 expression after stimulation with 10 ng/mL of lipopolysaccharide in RA patients (*r* = 0.057, *P* = 0.787).

As shown in Figure 4, HOMA index significantly correlated with fold increase of TLR2 expression (calculated as MFI) in monocytes of RA patients after stimulation with 10 *μ*g/mL peptidoglycan (*r* = 0.441, *P* = 0.027, Figure 4(a)), while failing to reveal significant correlation in normal controls (*r* = 0.249, *P* = 0.492, Figure 4(b)). Additionally, there was no significant correlation between HOMA index and TLR4 expression (calculated as MFI) after stimulation with 10 ng/mL lipopolysaccharide (*r* = 0.057, *P* = 0.787, Figure 4(c)).

### 3.4. Relationship between TNF-*α* Concentration and TLR2 Expression in Monocytes after Stimulation with Peptidoglycan in Normal Controls and RA Patients

**Figure 5:**
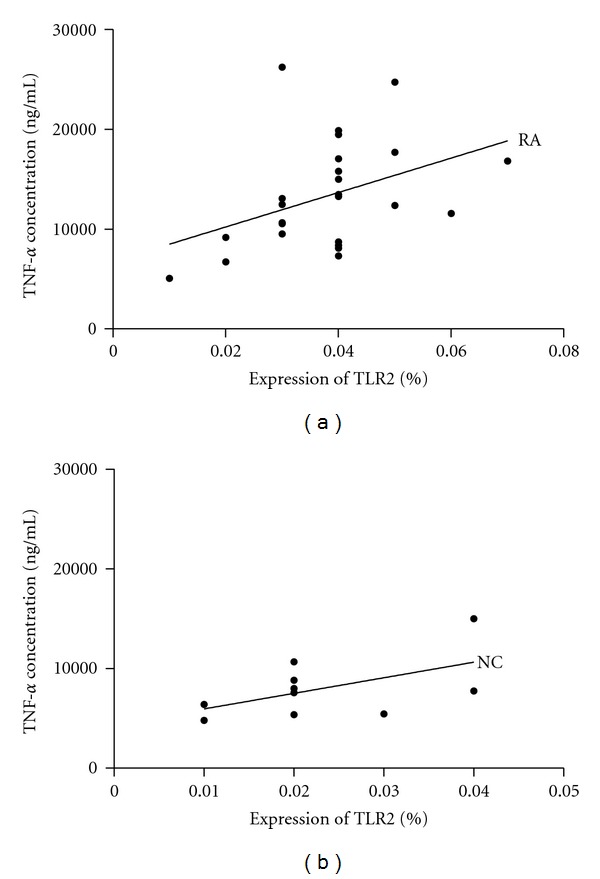
Relationship between TNF-*α* concentration and TLR2 expression (calculated as percentage of CD14^+^ monocytes expressing TLR2) in monocytes after stimulation with 10 *μ*g/mL of peptidoglycan in RA patients and normal controls (NC). (a) TLR2 expression significantly correlated with TNF-*α* concentration in RA patients (*r* = 0.484, *P* = 0.014); (b) no significant correlation in normal controls (*r* = 0.469, *P* = 0.172).

As shown in Figure 5, TLR2 expression (calculated as percentage of CD14^+^ monocytes expressing TLR2) significantly correlated with TNF-*α* concentration following stimulation with 10 *μ*g/mL of peptidoglycan (*r* = 0.484,  *P* = 0.014), while failing to reveal significant correlation in normal controls (*r* = 0.469, *P* = 0.172). In addition, HOMA index was significantly correlated with concentration of TNF-*α* (*r* = 0.433, *P* = 0.019) in RA patients.

### 3.5. Relationship between IL-6 Concentration and TLR2 Expression in Monocytes following Stimulation with Peptidoglycan in Normal Controls and RA Patients

**Figure 6:**
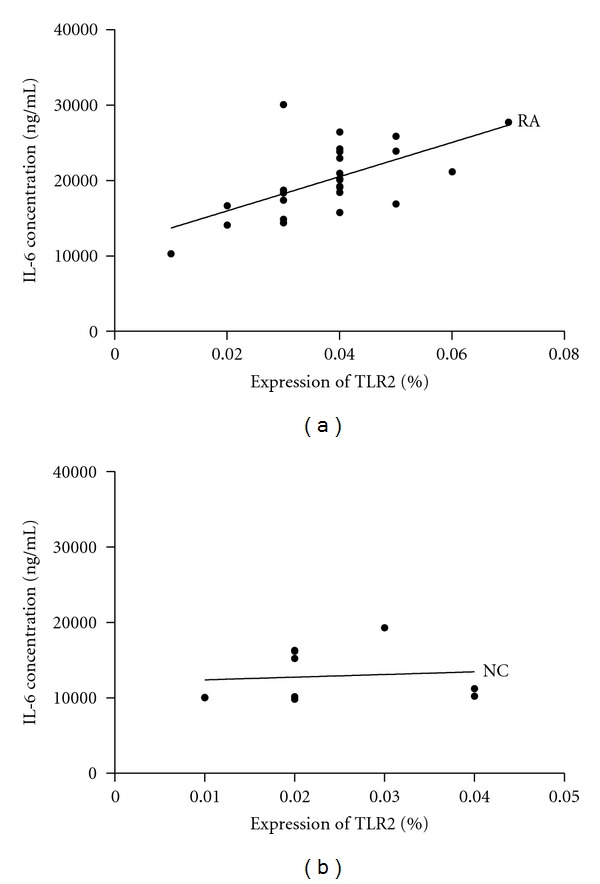
Relationship between IL-6 concentration and TLR2 expression (calculated as percentage of CD14^+^ monocytes expressing TLR2) in monocytes after stimulation with 10 *μ*g/mL of peptidoglycan in RA patients and normal controls (NC). (a) In RA patients, TLR2 expression was significantly correlated with IL-6 concentration (*r* = 0.611, *P* = 0.001); (b) no correlation in normal controls (*r* = 0.449, *P* = 0.193).

As shown in Figure 6, TLR2 expression (calculated as percentage of CD14^+^ monocytes expressing TLR2) was significantly correlated with IL-6 concentration following stimulation with 10 *μ*g/mL of peptidoglycan (*r* = 0.611, *P* = 0.001) in RA patients, but not significant in healthy volunteers (*r* = 0.449, *P* = 0.193). In addition, HOMA index was significantly correlated with concentration of IL-6 (*r* = 0.468, *P* = 0.009) in RA patients.

## 4. Discussion

The major finding of the present study is that TLR2 expression, after stimulation with peptidoglycan, showed significant correlation with insulin resistance in patients with RA in the absence of hyperglycemia. RA is one of the most prevalent autoimmune diseases and affects about 0.5–1% of the adult population. The hallmark of RA is persistent polyarticular synovitis mainly affecting the small joints. Patients with RA have increased morbidity and mortality from cardiovascular disease such as atherosclerosis compared to patients without RA [1]. Both systemic inflammation and insulin resistance are reported to be important players in the development of atherosclerosis [32]. Insulin resistance was considered to contribute to the increased cardiovascular risk in the general population [33, 34]. Inflammation has been identified as fundamental in insulin resistance in patients with RA [5, 35]. The inflammatory pathways can be integrated to cause insulin resistance by activating membrane receptors such as TLRs [12, 36, 37]. TLRs are critical in the recognition of invading pathogens and activation of subsequent immune responses against them. Upon stimulation, TLRs induce the activation of NF-*κ*B and mitogen-activated protein kinases (MAPK) and the expression of inflammatory cytokines [36, 38]. Palmitate treatment of differentiated C2C12 myotubes, through TLR2 activation, led to a time-dependent inhibition of insulin-activated signal transduction [21]. The inhibition of TLR2 expression can rescue cells from the activation of MAPK8 and improve insulin resistance [39]. TLR2 is crucial for diet-induced metabolic syndrome because mice lacking TLR2 are substantially protected from diet-induced adiposity and insulin resistance [40]. Additionally, polymorphisms in the TLR2 receptor gene have been linked to populations at high risk of developing type 2 diabetes [41, 42]. Overall, TLR2 could be a key modulator between inflammatory pathways and metabolic disorders such as insulin resistance.

Numerous factors are involved in the expression and activation of TLR2. Obesity and type 2 diabetes are associated with increased expression of TLR2 [43]. Obesity can induce increased c-Jun N-terminal kinase (JNK) activity [44, 45] which is activated in response to inflammatory cytokines, free fatty acids [22], activated NF-*κ*B, and inflammatory mediators, including TNF-*α* and IL-6, and may contribute to insulin resistance [23, 24]. Furthermore, raised free fatty acids and TG in obese individuals and animals can be important etiologies of insulin resistance [46]. High glucose induces inflammatory cytokines, chemokines, p38 MAPK, NF-*κ*B activity [47–52], and TLR2 expression [17]. Recently, Dasu and Jialal further indicated that free fatty acids in the presence of high glucose amplify monocyte inflammation via TLRs [18]. Collectively, TLRs inducing inflammatory pathway may contribute significantly to insulin resistance, particularly in the presence of obesity related conditions or high blood sugar. However, rare data exist describing the roles of TLRs in the development of insulin resistance in the absence of raised blood sugar in RA patients. The present study found that TLR2 expression in circulating CD14^+^ monocytes, after stimulation with peptidoglycan, correlated significantly with HOMA index in nondiabetic RA patients. Additionally, the expression of TLR2, upon stimulation with peptidoglycan, was correlated with levels of TNF-*α* and IL-6. HOMA index was also significantly correlated with concentration of IL-6 and TNF-*α* in RA patients. These data implied that TLR2 could contribute to the development of insulin resistance in patients with RA without concurrent hyperglycemia. Additionally, the significant correlations of the HOMA index with TG and ratio of TG to HDL in RA patients are consistent with the previous literature. Waist circumstance and BMI, as parameters measured while evaluating obesity, displayed a significant correlation with HOMA index in this study. Control of obesity thus may also be critical in treating RA patients at a high risk of insulin resistance. Further study is required to elucidate the detailed roles of peptidoglycan or other TLR2 ligands in the occurrence of insulin resistance. This study indicates that body mass index, waist, TG, and ratio of TG to HDL were significantly associated with HOMA index in nondiabetic RA patients. These findings are consistent with previous investigations showing that insulin resistance is associated with low HDL cholesterol and high TG in patients with inflammatory arthritis [53, 54]. Therefore, the so-called traditional risk factors also need to be closely monitored and carefully treated.

## 5. Conclusion

The results demonstrating the close relationship between HOMA index and TLR2 expression in monocytes and inflammatory cytokines such as TNF-*α* and IL-6 could provide therapeutic interventions against insulin resistance in nondiabetic RA patients. Treating traditional factors such as BMI, waist, TG, and ratio of TG to HDL cholesterol is crucial in minimizing the development of insulin resistance in RA patients, even in the absence of hyperglycemia. This study may lead to a better understanding of the relationship between TLR2 expression and insulin resistance in patients with RA. Further investigation is necessary to elucidate the role of TLR2 *in vivo*.

## Figures and Tables

**Table 1:** Demographic and clinical characteristics of normal controls and RA patients.

Characteristics	Normal controls^b^ (*n* = 10)	RA patients^b^ (*n* = 30)	*P* ^c^
Demographics			
Age (years)	57 (51–64)	57 (36–82)	0.778
Sex (percentage of females)	90	83	0.632
Cardiovascular risk factors			
Systolic blood pressure (mm Hg)	135 (91–170)	132 (89–170)	0.815
Diastolic blood pressure (mm Hg)	82 (60–105)	80 (51–107)	0.790
BMI^a^ (kg/m^2^)	24.9 (20.7–31.2)	24.3 (19.3–38.7)	0.373
Cholesterol (mg/dL)	197 (145–297)	196 (122–270)	0.755
Low-density lipoprotein (mg/dL)	111 (63–180)	104 (32–164)	0.719
High-density lipoprotein (mg/dL)	60 (36–80)	62 (35–104)	0.751
Triglycerides (mg/dL)	91 (36–178)	134 (48–566)	0.199
TG to HDL	1.7 (0.6–4.8)	2.6 (0.5–15.3)	0.325
TC to HDL	3.4 (2.3–4.8)	3.3 (2.2–4.8)	0.876
Glucose (mg/dL)	92 (82–104)	90 (66–115)	0.406
HOMA^a^	0.9 (0.3–2.2)	2.1 (0.3–13.0)	0.017
Measures of disease activity			
Disease activity (DAS28^a^)	NA^a^	4.9 (3.1–7.4)	NA^a^
Current use of corticosteroids, number (%)	NA^a^	25 (83)	NA^a^
Cumulative corticosteroids dose (gm)	NA^a^	2.7 (0.14–9.6)	NA^a^
Other markers of inflammation			
ESR^a^ (mm/h)	14 (3–29)	35 (5–80)	0.002
HSCRP^a^ (mg/L)	1.2 (0.2–3.4)	9.0 (1.0–57.5)	0.008

^a^Abbreviations: BMI: body mass index; DAS28: Disease Activity Score in 28 joints; ESR: erythrocyte sedimentation rate; HOMA: homeostasis model assessment; HSCRP: high sensitivity C-reactive protein; NA: not applicable; RA: rheumatoid arthritis.

^b^Data are expressed as mean and range.

^c^ *P* < 0.05 was considered statistically significant.

## Abbreviations

ESR: Erythrocyte sedimentation rate
HOMA: Homoeostasis model assessment
HSCRP: High sensitivity C-reactive protein
Ik*κβ*: Inflammatory kinase I-kappa-B kinase-*β*
IL-6: Interleukin-6
JNK: Jun N-terminal kinase
MAPK: Mitogen-activated protein kinases
MFI: Mean fluorescence intensity
NF-*κ*B: Nuclear factor-*κ*B
PBS: Phosphate-buffer saline
RA: Rheumatoid arthritis
TG: Triglyceride
TLR: Toll-like receptor
TNF-*α*: Tumor necrosis factor-alpha.
